# Effects of Exogenous Glucoamylase Enzymes Alone or in Combination with a Neutral Protease on Apparent Total Tract Digestibility and Feces *D*-Lactate in Crossbred Angus Bulls Fed a Ration Rich in Rolled Corn

**DOI:** 10.3390/ani10061077

**Published:** 2020-06-23

**Authors:** Maria Devant, Shukun Yu, Sandra Genís, Torben Larsen, Li Wenting

**Affiliations:** 1 Ruminant production, IRTA, Torre Marimon, 08140 Caldes de Montbui, Spain; sandra.genis@gmail.com; 2 DuPont Nutrition & Biosciences, Edwin Rahrs Vej 38, 8220 Brabrand, Denmark; Shukun.Yu@dupont.com; 3 BonÀrea Agrupa, Traspalau 8, 25210 Guissona, Spain; 4 Department of Animal Science, Blichers Allé 20, 8830 Tjele, Denmark; torben.larsen@anis.au.dk; 5 DuPont Nutrition & Biosciences, Wilmington, DE 19803, USA; WENTING.LI@dupont.com

**Keywords:** beef, feed, glucoamylase, protease, total tract digestibility, fecal *D*-lactate

## Abstract

**Simple Summary:**

Dietary enzyme supplementation, as a feed additive, has been well adopted in monogastric production to increase feed efficiency. However, in ruminants, considerably fewer studies have been done and fewer, if any, commercial enzymes have been adopted as a feeding strategy. Feedlot cattle are commonly fed high-starch diets, with varying starch digestibilities depending on type of grain, and degree of grain processing. Improvement in starch digestibility in low processed grain diets will undoubtedly warrant economic benefits to feedlot producers and reduce environmental impact of intensive beef production. In this study, we have shown that dietary glucoamylase supplementation improved 7 to 13% apparent digestibility of dry matter and starch in bulls fed rolled corn-based diets, suggesting that enzyme (glucoamylase) supplementation could be a promising strategy to improve starch efficiency for finishing beef cattle.

**Abstract:**

The aim of this study was to evaluate the effect of two glucoamylases (GA) and the combination of one GA with a neutral protease on apparent total tract digestibility in beef bulls fed a total mixed ration (TMR) rich in rolled corn. Sixteen Angus beef bulls (266 ± 4.9 kg of initial BW, and 182 ± 1.7 d of age) were distributed in 4 blocks, each block consisted of 4 animals balanced by BW. The experimental design was a 4 × 4 Latin square (4 blocks and 4 periods, 2 w per period). Four treatments were tested; (1) control, (2) GA preparation from *Trichoderma reesei* (TrGA); (3) GA from *Aspergillus fumigatus* (AfuGA); (4) AfuGA in combination with a neutral protease from *Bacillus amyloliquefaciens* (BamPro). Apparent total tract digestibility and fecal *D*-lactate concentration were analyzed. Enzyme supplementation, regardless of enzyme type, increased apparent total tract digestibility of dry matter (from 66.7% to 73.1% ± 2.01), and starch (from 74.7% to 81.8% ± 2.25), without affecting feces *D*-lactate concentration. Irrespective of glucoamylase type, glucoamylase supplementation improved apparent digestibility of dry matter and starch, and the addition of a protease did not have additional benefits on nutrient digestibility.

## 1. Introduction

Energy intake and digestibility, particularly the energy obtained from starch, is the limiting factor for growth in feedlot beef cattle [1]. Corn is the predominant grain (dry rolled, ground, high moisture or steam flaked) and starch source used in the US, Central and Latin America feedlots [2]. Among the grain processing methods, steam flaking is an effective way to increase starch availability for ruminants. Studies have shown that feeding steam flaked corn in finishing cattle increased ruminal and total tract starch digestibility about 10 to 20% and 7 to 10%, respectively, compared with dry rolled corn [3,4]. However, steam flaking is a process that requires large capital investment, and thus might be cost prohibitive for small farms. Therefore, alternative methods to enhance the starch availability for dry rolled corn will be beneficial to producers, especially when access to steam flakers is limited. Unfortunately, given the amount of research done on enzyme application in ruminants, industry-wise adoption of exogenous enzyme is still very low, partly due to inconsistent results. In addition, the majority of the enzyme application studies were done with fiber-degrading enzymes, such as xylanase, and information on starch degrading enzyme is sparse. The present study is one of the first studies where glucoamylases (GA) from glycoside hydrolase family 15 (GH15) (EC 3.2.1.3) are tested in cattle. The end product of these GA enzymes is glucose instead of the mixture of glucose, maltose and maltooligosaccharides which are the end products produced by α-amylase (AA) (GH13) (EC 3.2.1.1). Based on the abundance of genes, coding for the enzymes in the rumen [5,6] AA activity is much greater compared with GA. Therefore, it is hypothesized that supplementing ruminally active GA will complement AA activity and enhance starch degradation as a result. With that, the current study evaluated the efficacy of two GAs, TrGA (from *Trichoderma reesei*) and AfuGA (AfuGA from *Aspergillus fumigatus*) in beef cattle fed corn-based, high concentrate finishing diet. Both enzymes were active in the rumen and highly specific to maltose, as observed in previous in vitro studies [7]. In addition, a neutral protease (BamPro from *Bacillus amyloliquefaciens*; [8]) stable at rumen pH (from 5.5 to 7) was included as a third treatment and supplemented in combination with AfuGA (AfuGA+BamPro), hypothesizing that the combination would synergistically increase starch digestibility with GA by degrading the protein matrix encasing starch granule in the endosperm [9]. Therefore, the novelty in the present study is that, as mentioned previously, it differs from most previously published studies where fungal and bacterial AA and bacterial serine protease have been evaluated [10,11]. Finally, decreased *D*-lactate concentrations in feces could be an indicator of improved overall starch digestion in the digestive tract, since *D*-lactate is of microbial origin and not produced by mammalian cells [12]. The objective of the present study was to evaluate the effect of two exogenous GA enzymes, alone or in combination, with a neutral metalloprotease on apparent total tract digestibility and on fecal *D*-lactate concentration in crossbred Angus bulls fed a high rolled corn-based diet.

## 2. Materials and Methods

### 2.1. Animals, Housing, Experimental Design and Diets

All experimental protocols were approved by the Institutional Animal Care Committee of the Institut de Recerca i Tecnologia Agroalimentàries (Barcelona, Spain, number FUE-2018-00702882- 9970), and the study was conducted in accordance with the Spanish guidelines for experimental animal protection [13].

Sixteen Angus beef bulls (266 ± 4.9 kg of initial BW, and 182 ± 1.7 d of age) were allocated in individual, partially slatted pens (1.9 × 3.4 m) at the experimental station of the Cooperativa Agraria de Guissona (Guissona, Lleida, Spain). Before starting the study, all animals were adapted to the experimental total mixed ration (TMR); from −14 to −11 day all animals were fed with concentrate (32.9% rolled corn, 11.5% wheat middlings, 10.0% hominy feed, 9.5% barley, 8.0% corn dried distilled grains, 10.0% soybean meal, 7.0% wheat, 3.0% sunflower meal, 1.5% beet pulp, 1.0% palm oil, 3.46% vitamin-mineral premix, 1.77% calcium carbonate, 0.37% salt, 3.25 Mcal/kg ME, 16.5% Crude Protein (CP), 5.6% ether extract, 17.2% NDF and 5.9% ash) and long straw (3.5% CP, 1.6% ether extract, 70.9% NDF, and 6.1% ash; DM basis), both ad libitum, in two separate feeders (0.6 × 1.2 × 0.3 m). From −10 to −6 day animals were fed with concentrate and long straw in two separate feeders (0.6 × 1.2 × 0.3 m), and TMR (see Table 1), in a separate feeder (0.6 × 1.2 × 0.3 m), free of choice and ad libitum; from −5 to day 0 all animals were fed TMR only ad libitum. The TMR was the same in all treatments and was formulated according to NRC [14] recommendations. During the study, the TMR was fed in one trough (0.6 × 1.2 × 0.3 m) and all animals were fed ad libitum. The pens were also equipped with a water bowl drinker.

Animals were assigned to one of the four blocks, each block had 4 animals, and blocks were balanced by animal BW. The experiment was designed as a 4 × 4 Latin Square with blocks and periods of 2 w. The total study length was of 8 w. Each block in the first period was randomly assigned to 1 of 4 treatments. The four tested treatments were: Treatment 1 (CTR) was blank solution where the solution was mixed with the total mixed ration (TMR) at a rate of 10 mL for 40 kg TMR. The blank solution contained 0.2% potassium sorbate and 0.6% sodium benzoate that corresponded to the preservatives used in the liquid enzyme preparations; Treatment 2 was a GA preparation from the fungus *T. reesei* (TrGA); Treatment 3 was a GA preparation from *A. fumigatus* (AfuGA); Treatment 4 (AfuGA+BamPro) was AfuGA and a *B. amyloliquefaciens* neutral protease (EC 3.4.24.28) preparation (BamPro). TrGA and AfuGA were dosed at 150 and 100 glucoamylase units per kg TMR, respectively, measured by using 4-nitrophenyl-α-*D*-glucopyranoside as substrate at pH 4.3 and 30 °C. The dose for the protease was 39,100 protease units/kg TMR. All the enzymes were mixed with the same blank solution as CTR and were provided by DuPont Nutrition & Biosciences Aps (Brabrand, Denmark). Enzyme preparations were kept at 4 °C and were mixed daily with the TMR 15 min before animals were fed.

### 2.2. Measurements and Sample Collection

Health status (presence of coughing, visible discharge in nose or eyes, droopy ears, head tilt, diarrhea, bloat and fever) was recorded daily.

#### 2.2.1. Feed Consumption

Feed offers (concentrate and straw or TMR) were recorded daily at 9:00 a.m., and feed leftovers were registered daily at 8:00 a.m. Animal BW was recorded every two weeks at the beginning of each period.

#### 2.2.2. Apparent Total Tract Digestibility and *D*-lactate Concentration in Feces

Two criteria were chosen to determine the suitable duration of the experimental period (14 days). First, it should be sufficient to avoid carry-over effects from previous treatments (washout). Second, this period should be as short as possible to reduce the risk that the animals may drastically change their rumen fermentation, metabolism and intake during the study because these animals were still growing. Considering that the basal-high-concentrate diet remained unchanged during the entire study and that research indicates that ruminant total tract digestibility ordinarily adjusts to diet within 10 to 14 d when high concentrate diet (>50%) is fed [15,16], the adaptation period was set at 12 days for the current study. From day 8 to 14 chromium oxide (1 g/kg DM) was added to the TMR as an indigestible marker for nutrient digestibility determination. During these days a sample of feed offer (concentrate and mixed ration) and refusals from each animal were collected. Fecal grab samples were collected from day 12 to 14 from the rectum and dried at 55 °C for 48 h, and composited by animal and period on an equal DM basis.

#### 2.2.3. Chemical Analyses

Feed samples of each dietary treatment were collected every 2 weeks for determination of nutrient composition. Samples were analyzed for DM (24 h at 103 °C; method number 925.04; [17]), ash (4 h at 550 °C; method number 642.05; [17]), CP by the Kjeldahl method (method number 988.05; [17]), NDF according to Van Soest et al. [18] using sodium sulfite and AA and fat using a Soxhlet apparatus after an acid hydrolysis preparation [17]. Total starch content was analyzed using the polarimetric method according to the EU Regulation for feed analyses (n° 152/2009). Chromium concentration of feed and fecal samples were determined based on the procedure of Le Du and Penning [19]. Digestion was carried out on duplicates weighing 0.5 g of sample. Two digestion steps were made. The first digestion step was performed with 4 mL HNO_3_ (65%, *w*/*w*) concentrated at 220° during 15 min, in a microwave oven (Ultrawave model, Milestone, Sorisole, Italy); uncolored solutions were obtained with a green solid at the bottom of the digestion tube. That solid is attributed to Cr_2_O_3_(s). In the second step, 3 mL of H_2_SO_4_ (95–97%, *w*/*w*), 0.5 mL of HClO_4_ (32%, *w*/*w*) and 2 mL of hydrofluoric acid (40%, *w*/*w*) at the same digestion tube were added and new a digestion procedure was made at 260 °C for 15 min. Finally, the Cr content was determined by inductively coupled plasma optical emission spectrometry (model Optima 4300D, Perking-Elmer, Shelton, CT, USA).

*D*-lactate concentration in deproteinized feces was analyzed by an enzymatic-fluorometric method, according to Larsen [20]. Dried feces (200 mg) were extracted for 2 h in 2.00 mL 66% (V/V) ethanol, at room temperature with periodic vibrations on a whirl mixer. Samples were centrifuged for 10 min (3500× *g*) and supernatants obtained were used for analyses after diluted with 66% ethanol.

### 2.3. Calculations and Statistical Analyses

Apparent total tract digestibility was calculated estimating total fecal output, which was estimated as the ratio of chromium intake to chromium concentration in the feces.

Data were analyzed using a mixed-effects model (Version 9.2, SAS Inst., Inc., Cary, NC, USA). The model included initial BW as a covariate, treatment, period (14 d period), as fixed effects, and block and animal nested within block as random effect. Differences between treatments were compared using the PDIFF option in the LSMEANS statement. For all analyses, significance was declared at *p* ≤ 0.05 and tendencies were discussed at 0.05 < *p* ≤ 0.10.

## 3. Results and Discussion

Throughout the study, there were no health incidences recorded and treatment did not affect total feed intake (Table 2). Apparent total tract digestibility values were slightly lower than, but still within, the observed range when bulls are fed high-concentrate diets rich in rolled corn [21,22].

The current study is the first attempt to evaluate GA, alone or in combination with protease on starch digestibility in large ruminants. Glucoamylases were selected in this study considering rumen microbiota metagenome data available in recent years showing more abundancy of AA genes than GA genes [5,6]. In the current study, TrGA and AfuGA significantly improved apparent total tract starch digestibility by 7 and 13%, respectively, as compared with control CTR (*p* < 0.05). Rojo et al. [23] reported in lambs that feeding sorghum grain with GA from *A. niger* did not affect ruminal or total tract starch digestion but resulted in higher partial feed efficiency. Mota et al. [24] supplemented the same GA to lambs fed corn-based high concentrate diets and found significant improvement in feed conversion ratio (12%) as compared with those fed a non-enzyme control diet, with similar average daily gain. It is not easy to attribute the exact cause(s) for observing different responses between lamb and cattle (current study), but feeding frequency, animals’ physiological status and enzyme inclusion ratio, application method, impurities in the enzyme mixture, degree of grain processing, etc., could be part of the explanation [25,26,27].

Contrary to our hypothesis [26,28], supplementing GA with protease showed no synergic effect on any of the parameters tested. Several studies have reported the effects of various proteases on digestibility in ruminants. In dairy cows, Eun and Beauchemin [29] studied the effects of exogenous proteolytic enzymes (serine protease type) in lactating Holstein cows fed high or low forage diets and observed an increased starch digestibility in enzyme-supplemented treatments when cows were fed high-forage diets. In growing beef cattle [11], the addition of an experimental exogenous proteolytic enzyme increased DMI of steers by 14.8%, but reduced NDF digestibility (4.1%), but no impact on performance (weight gain or feed efficiency). In finishing cattle, no effects were observed on digestibility or performance when the same enzyme was supplemented [11].

**Figure 1:**
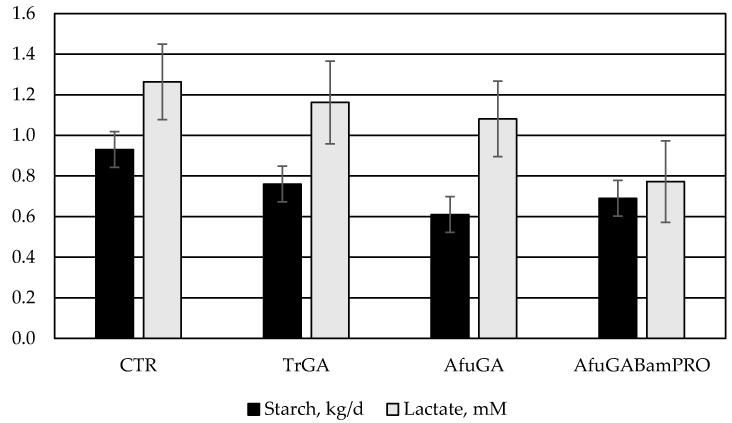
Daily fecal starch output (kg/d) and fecal *D*-lactate concentration (mM) in crossbred Angus bulls fed TMR supplemented with the different enzymes (TrGA = *Trichoderma reesei* glucoamylase; AfuGA = *Aspergillus fumigatus* glucoamylase; AfuGABamPro = *Aspergillus fumigatus* glucoamylase + *Bacillus amyloliquefaciens* neutral protease).

*D*-lactate is of microbial origin and not produced by mammalian cells. *D*-Lactate concentration in feces is a resulting product of fermentation of carbohydrates in the intestine, particularly the large intestine [12]. Therefore, decreased *D*-lactate concentrations in feces could be an indicator of improved overall starch digestion in the digestive tract. In feedlot cattle, decreasing feces *D*-lactate and fecal starch content may decrease the risk of hind-gut acidosis. Therefore, exogenous enzymes could also improve animal performance by not only enhancing ruminal carbohydrate degradability but also postruminal nutrient absorption and fermentation [30]. In the present study, feces *D*-lactate concentration followed a similar pattern to feces starch daily excretion (Figure 1), even though the results were not significant (*p* = 0.29).

## 4. Conclusions

In summary, in crossbred Angus fed over 70% of rolled corn, apparent total tract starch digestibility was significantly increased when supplementing a fungal glucoamylase from *T. reesei* or *A. fumigatus* or a mixture of the *A. fumigatus* glucoamylase and a neutral protease from *B. amyloliquefaciens*.

## Figures and Tables

**Table 1:** Ingredient and nutrient composition of total mixed ration (TMR).

Item	
Ingredients, %	
Rolled corn	70.0
Corn DDG	15.0
Alfalfa hay chopped at 3–5 cm	10.0
Soybean meal 44% CP	2.40
Calcium carbonate	1.00
Bicalcium phosphate	0.40
Sodium bicarbonate	0.40
Urea	0.30
Salt	0.30
Vitamin-mineral premix ^1^	0.20
Nutrients, % DM basis	
ME, Mcal/kg	3.19
CP, %	16.7
Ether extract, %	6.2
NDF. %	21.6
Starch, %	48.0
Ash	5.1
Calculated non-structural carbohydrate	50.4

^1^ Nucleous for finisher concentrate (CAG, Guissona, Spain): vitamin and mineral contained per kg of DM: 3575.8 kIU of vitamin A, 858.6 kIU of vitamin D_3_, 101 g of vitamin E, 2.3 g of vitamin B_1_, 0.2 g of Co, 2.5 g of Cu, 0.26 g of I, 15.7 g of Mn, 0.15 g of Se, 20.6 g of Zn, 7.2 g of Fe, 75.8 g of etoxiquine and 1 kg of barley as excipient. 3. Metabolizable energy(ME).

**Table 2:** Daily nutrient intake, nutrient fecal output and apparent total tract digestibility in crossbred Angus bulls fed TMR supplemented with the different enzymes (TrGA = *Trichoderma reesei* glucoamylase; AfuGA = *Aspergillus fumigatus* glucoamylase; AfuGABamPro = *Aspergillus fumigatus* Glucoamylase + *Bacillus amyloliquefaciens* neutral protease).

	Treatment ^1^					
	CTR	TrGA	AfuGA	AfuGA + BamPro	SEM	*p*-Value ^2^
Intake, kg/d						
DM	7.8	7.9	8.0	7.6	0.12	0.19
OM	7.4	7.5	7.6	7.3	0.12	0.18
Starch	3.7	3.8	3.8	3.7	0.07	0.29
CP	1.3	1.3	1.3	1.2	0.03	0.11
Ether extract	0.49	0.49	0.49	0.46	0.009	0.10
NDF	1.7	1.7	1.7	1.7	0.03	0.33
Fecal output, kg/d						
DM	2.5	2.3	2.0	2.1	0.16	0.15
OM	2.4	2.1	1,9	1.9	0.16	0.15
Starch	0.93	0.76	0.61	0.69	0.088	0.07
CP	0.40	0.37	0.35	0.35	0.027	0.45
Ether extract	0.18	0.18	0.17	0.17	0.013	0.86
NDF	0.79	0.76	0.72	0.71	0.050	0.61
Apparent total tract digestibility, %						
DM	66.7 ^b^	71.1 ^a^	74.7 ^a^	72.8 ^a^	2.01	0.05
OM	66.8 ^b^	71.3 ^a^	74.9 ^a^	72.9 ^a^	2.03	0.05
Starch	74.7 ^b^	80.2 ^a^	84.1 ^a^	81.3 ^a^	2.25	0.04
CP	67.7	70.8	74.4	72.4	2.14	0.17
Ether extract	61.2	62.5	64.7	62.7	2.87	0.84
NDF	50.0	53.9	58.4	58.1	2.91	0.15

^1^ CTR = no supplementation, only preservatives, TrGA = *Trichoderma reesei glucoamylase*; AfuGA = *Aspergillus fumigatus glucoamylase*; AfuGABamPro = *Aspergillus fumigatus glucoamylase* + *Bacillus amyloliquefaciens neutral protease*). ^a,b^ Within a row means with different superscripts are statistically different (*p* < 0.05). ^2^ Treatment effect. organic matter (OM).
